# Perceived knowledge of scheme members and their satisfaction with their medical schemes: a cross-sectional study in South Africa

**DOI:** 10.1186/s12889-022-14106-8

**Published:** 2022-09-08

**Authors:** Francis M’bouaffou, Eric Buch, Steve Olorunju, Evelyn Thsehla

**Affiliations:** 1 School of Health Systems and Public Health, University of Pretoria, Pretoria, South Africa; 2 Biostatistics Unit, South African Medical Research Council, Pretoria, South Africa; 3 SAMRC/WITS Centre for Health Economics and Decision Science, School of Public Health, Faculty of Health Sciences, University of the Witwatersrand, Johannesburg, South Africa

**Keywords:** Medical schemes, Knowledge, Prescribed minimum benefits, Member satisfaction, Knowledge

## Abstract

**Background:**

South Africa has a dual healthcare system comprising of private and public sectors covering 16% and 84% of the population, respectively. Medical schemes are the primary source of health insurance in the private sector. The aim of this study was to assess members of medical schemes' perceived knowledge and satisfaction with their medical schemes.

**Methods:**

A cross-sectional survey was conducted using a stratified systematic sample of members of 22 open medical schemes. Medical schemes members completed an online questionnaire on knowledge and satisfaction with their medical schemes. We calculated a composite perceived knowledge and satisfaction score. Descriptive, bivariate and multivariate analysis was conducted.

**Results:**

A total of 336 members of medical schemes participated in this study. Respondents generally perceived themselves to have good knowledge of their medical schemes. Eighty-one percent of participants were satisfied with the quality of services received from their designated service providers (DSPs), however, only 9% were satisfied with accessibility of doctors under their DSP arrangement. Twenty-five percent of respondents were satisfied with scheme contributions and only 46% were satisfied with the prescribed minimum benefit package.

**Conclusion:**

Medical schemes remain a key element of private healthcare in South Africa. The analysis shows that medical schemes, should put more effort into the accessibility of general practitioner under their designated service providers. Furthermore, the prescribed minimum benefits should be reviewed to provide a comprehensive benefits basket without co-payment for members as recommended by the Medical Schemes Act Amendment Bill of 2018.

**Supplementary Information:**

The online version contains supplementary material available at 10.1186/s12889-022-14106-8.

## Background

South Africa has a dual healthcare system, with private and public sectors covering 16% and 84% of the population, respectively [1–3]. The two sectors operate in parallel in a national health system that faces strong complaints about healthcare inequity and patient satisfaction [1–4]. In South Africa, health care is financed through general tax revenue, medical schemes contributions, and out-of-pocket payments (OOPs). The general taxes fund the public sector while the private insurance funds the private sector. Private insurance funds are called medical schemes and are the primary source of health financing in the private sector [5] medium [5]. Medical schemes offer voluntary pre-payment and are utilized to access healthcare in the private health sector. There are significant gaps in terms of coverage and access to health care services in the tax-funded public sector and the services offered in the private sector through medical schemes, particularly the lower cost options [3, 6].

In 2017, there were 82 medical schemes consisting of 22 open schemes and 60 restricted schemes [7]. *Open Schemes* are open to any applicant referred to as a principal member. A principal member of a medical scheme is a person responsible for paying contribution(s) to the medical scheme. Principal members may register dependants on the schemes according to the scheme rules. Members and dependants are both named beneficiaries of the scheme. A principal member of a scheme can be any person above 18 years, not a member of any other medical scheme, and be able to pay the monthly contributions [7, 8]. *Restricted Schemes* limit their membership using specific criteria, such as a profession, an employer group, or a commercial or industrial sector [5, 7].

There are significant differences between the two in terms of demographics, number of beneficiaries and the range of benefit options they offer. In 2018, the benefits options in open schemes registered with the Council for Medical Schemes (CMS) was 181 as compared to 143 in restricted schemes [8]. The high number of health plans in open schemes have been identified as a challenge as members cannot identify those that offer the best value for money [3–5].

Furthermore, medical schemes offer benefits that are usually not comprehensive, leading to out of pocket payments for services not covered by schemes [1, 6]. In 2015, OOP was estimated to be 0.6% of GDP [4]. According to Mohammed and Dong, beneficiaries’ complaints rise when providers deprive enrolees of their full entitlements or when additional fees are added [9]. Despite these challenges, only one independent survey of medical scheme members’ knowledge, attitudes and perceptions has been conducted [10]. This study found that 76% of respondents understood the cost implication and benefits options of the medical schemes and had good accessibility to a private doctor or hospital.

In Nigeria, a study on knowledge, attitude, and perception (KAP) has shown that people have great expectations for their schemes. Older individuals were generally more knowledgeable about insurance as were males and those enjoying a better education [11]. In another Nigerian study, satisfaction rate was rated high (42%) for enrolees in a scheme with length of employment, salary income, hospital visits and duration of enrolment shown to slightly influence satisfaction [9]. In Nigeria and India, consumers did not always receive the information necessary to make informed benefit option choices and many were not aware of the publicly available information [9, 12].

The objective of this study is therefore to evaluate the perceived knowledge and satisfaction of open schemes members with their current medical scheme and to assess its association with socio-demographic factors and members' medical history.

## Methods

### Study method

We conducted a descriptive online cross-sectional survey of the principal members of open medical schemes in South Africa. We used a Google form to conduct the survey.

### Target population

The study population consisted of 2 347 757 open scheme principal members [7].

### Sample size

Based on a 5% margin of error, 95% confidence interval and an estimated response rate of 40%, the estimated sample size was 384. A stratified systematic sample with a random starting point was drawn from the members of the 22 open medical schemes in proportion to their size.

### Study setting

The study was conducted in the Republic of South Africa. In mid-2016 its population was estimated at 55,91 million inhabitants. South Africa is a multi-ethnic society with nine provinces and eleven official languages.

### Data collection method

The study team developed, piloted, and calibrated a purpose-specific questionnaire to evaluate the perceived knowledge and satisfaction of open schemes’ principal members. Twenty members were selected for the pilot study. Great care was taken in the phrasing of questions to avoid leading questions, or questions difficult to understand.

The medical schemes were briefed and their support for the survey was obtained. The medical schemes agreed to distribute the questionnaires to their members with a covering letter that briefly explained the purpose of the survey. The letter provided a Uniform Resource Locator to the informed consent form that simplified the use of the online questionnaire.

Primary data was collected through a structured questionnaire developed in conjunction with the CMS and sent via a Google form. The questionnaire comprised of open-ended and close-ended questions grouped within eight sections related to the medical scheme membership, general experience of the medical scheme, brokers, benefits option, prescribed minimums benefits (PMBs), designated service providers, complaints and appeals and lastly socio-demographic information.

The Council for Medical Schemes granted permission to conduct the research. The Research Ethics Committee of the Faculty of Health Sciences, University of Pretoria, approved the study.

### Data analysis

We conducted descriptive statistical analysis using frequency tables. We measured the scores of principal members’ level of satisfaction, and we compared them across factors such as socio-demographic characteristics.

Responses were on a 5-point scale of very positive, positive, neutral, negative and very negative. For statistical analysis, these were converted to numbers, where very positive was scored as a 5 and very negative as a 1. All responses coded 4 and 5 were recoded as 1, while codes 1, 2 or 3 were considered as 0 implying not a good perceived knowledge or satisfaction. Thereafter a score for each was computed by evaluating the performance of each respondent. The responses were recoded into three, namely good, average and poor to determine statistical association.

A score above 60^th^ percentile for perceived knowledge and 60^th^ for perceived satisfaction were considered better-perceived knowledge and good satisfaction with their schemes.

We tested for differences in the proportions of those with and without good perceived knowledge and satisfaction for different variables using the t-test for testing differences between proportion while Chi square the Fischer’s exact was used to assess the association between factors and outcome of interest and the Kruskal–Wallis rank tests was used to compare the responses of the degree of satisfaction by demographic factors.

We further calculated the odds ratio by constructing a binary outcome from the scores computed for satisfaction and knowledge. Satisfaction was classified as inadequate (0) if respondents scored less than 60% and categorized as adequate (1) where respondents scored 60% and above in the perception scale. Knowledge was categorised as good (1) for respondents who scored 80% and above in the knowledge score and poor (0) for those who scored less than 80%. A logistic regression was undertaken to assess the association between the outcome variables with socio-demographic factors and members' medical history.

## Results

### Socio-demographic characteristics

Although a sample size of 384 was planned, 336 (87%) members responded. Most respondents (73%) had tertiary education, 54% were married, 27% were 40–49 years old and 38% of respondents earned R30 000 (2 160 US$) or more. Seventy-two per cent regarded their overall health status as healthy, while 42% had a chronic disease. The top four chronic diseases were hypertension, diabetes, depression, and thyroid conditions. Thirty percent of respondents were on a low-level option, 55% on a medium option and 14% on a high option. The results are shown in Table 1.

**Table 1:** Socio-demographic characteristics of participants

**Variables**	**Frequency (%)**	
**Gender**		
Male	138(41)	
Female	198(59)	
**Age**		
20 – 29	29(9)	
30 – 39	83(25)	
40 – 49	90(27)	
50 – 59	65(19)	
≥ 60	66(20)	
**Marital status**		
Married	181(54)	
Single	91(27)	
Divorced	40(12)	
Others^a^	24(7)	
**Level of education**		
**No formal schooling**	1(0)	
**Primary school**^**b**^	1(0)	
Tertiary school	245(73)	
Secondary school^c^	87(26)	
**Monthly income**		
< R 5000	13(4)	
R5 000—< R10000	40(11)	
R10000—< R15000	50(15)	
R15000—< R25000	77(23)	
R25000—< R30000	27(8)	
≥ R30000	128(38)	
**Health status**		
Healthy	154(46)	
Excellent health	85(25)	
Moderately healthy	82(25)	
Poor health	12(4)	
Not Specified	3(1)	

^a^ Widowed, living with partner,

^b^ Grade 1 to Grade 7,

^c ^Grade 8 to12R1 = 0.072 US$ (2019)

### Principal members knowledge and satisfaction

Principal members were asked about their perceived knowledge of and satisfaction with their medical schemes, designated service providers (DSPs), brokers and drug coverage under the PMBs. Fifty seven percent of members considered their knowledge to be good and 66% were satisfied with the service provided by their scheme, whereas only 46% of respondents were satisfied with the PMB package.

Table 2 shows that 43% of participants had poor knowledge regarding the financial contributions they made to their medical scheme while 62% of respondents perceived their brokers to be knowledgeable about medical schemes. Eighty-one percent of respondents reported good satisfaction with the quality of services under the DSPs while 44% were satisfied with their prescription drug coverage under the PMBs. In terms of the DSPs, only 9% of the respondents were satisfied with their access to a DSPs general practitioner (GP). The principal members’ cumulative score for perceived knowledge was 67% whereas their satisfaction averaged 53%. Sixty percent of respondents were likely to recommend their medical scheme to others. Among the respondents, only 23% had ever lodged a complaint with their schemes; 26% of whom were satisfied with the outcome of their complaint. Twelve percent of them laid a complaint with the CMS and only 33% were satisfied with the outcome.

**Table 2:** Perceived knowledge and satisfaction of principal members

Variables	Frequency (%)		
	**Good**	**Average**	**Poor**
**Perceived Knowledge**			
Information received before joining scheme	190(57)	97(29)	46(14)
Understanding of benefits & costs	173(52)	100(30)	60(18)
Financial contribution made	83(25)	107(32)	143(43)
Brokers’ knowledge	206(62)	74(22)	54(16)
Knowledge about PMBs	181(54)	108(32)	47(14)
Knowledge about the DSPs	18,455	7422	77(23)
**Satisfaction**			
Satisfaction with brokers	191(57)	71(21)	74(22)
Interaction with scheme	218(65)	81(24)	37(11)
Service provided by scheme	222(66)	67(20)	14(14)
Satisfaction with the PMBs package	155(46)	104(31)	77(23)
Coverage of drug prescription under PMBs	148(44)	97(29)	91(27)
Quality of service under DSPs	272(81)	54(16)	10(3)
Accessibility to GP when needed	30(9)	54(16)	252(75)
Accessibility to medical specialist when needed	41(18)	64(19)	212(63)

### Factors that influenced the choice of a benefit option

When asked about the factors that influenced the choice of benefits option, the cost factor was shown to be the main factor driving the choice. The results showed that members chose an option based on what they can afford more than the benefits they felt they needed. Members sought an option that covered their chronic medication and provided adequate cover for their family, comprehensive hospital cover and/or cover for PMBs. However, for participants with chronic conditions, benefits influenced the choice of a scheme option. The cost of the premiums was also the key reason why members had changed their option. Table 3 shows that 54% of the participants changed their option due to the cost of their premiums compared to 17% due to employment and 29% due to other factors, mostly related to the participants’ health status.

**Table 3:** Reasons why members changed their benefit option

Variables (*N* = 145)	Frequency (n)	Frequency (%)
Premiums too expensive	78	54
Change of employment	24	17
Others (mostly health-related)	43	29

### Factors associated with perceived knowledge and satisfaction

Fischer’s exact test showed that there was a strong association between perceived knowledge and satisfaction (*p* = 0.001). Good satisfaction with the medical scheme was related to good perceived knowledge. Fischer’s exact test showed that there was no significant difference between good or poor perceived knowledge or satisfaction by gender, level of education or chronic disease. There was however a strong relationship between number of years on a medical scheme and good perceived knowledge *(pr* = 0.034). Good perceived knowledge and satisfaction were associated with a lesser number of years on the scheme (*pr* = 0.034). Good perceived knowledge and satisfaction were only found in those with 10 years or less of their scheme’s membership.

Members who joined their schemes through brokers had no better-perceived knowledge (*p* = 0.396). The result showed a marginal relationship between income and good perceived knowledge (*pr* = 0.093). However, there was a significant association between income and satisfaction (*pr* = 0.045). Good satisfaction of medical schemes was associated with high income. There was also a strong association between knowledge of the PMBs and general knowledge of the medical schemes (*p* = 0.053). Better perceived knowledge of the PMBs is associated with good general perceived knowledge. Fisher’s exact test showed that there was a significant relationship between good perceived knowledge and laying a complaint (pr = 0.006).

The results of the logistic regression however showed that only health status had a significant positive impact on perceived knowledge of a medical scheme. The results are shown in Table 4.

**Table 4:** Factors associated with perceived knowledge and satisfaction with a medical scheme

	**Un-adjusted Odd Ratio ± S.E**			**Adjusted Odd Ratio ± S.E**		
**Assessment of Perceived Knowledge**						
**Factors**	**Odd Ratio**	**95% C.I**	**P**	**Odd Ratio**	**95% C.I**	**P**
Gender	1.30 ± 0.30	[0.8 2.2]	0.33	0.70 ± 0.23	[0.36 1.35]	0.29
Age group	0.89 ± 0.09	[0.7 1.1]	0.27	0.83 ± 0.14	[0.83 1.78]	0.31
Marital Status	1.02 ± 0.04	[0.9 1.1]	0.52	1.04 ± 0.05	[0.94 1.57]	0.41
Education	1.28 ± 0.40	[0.7 2.30	0.43	0.69 ± 0.28	[0.32 1.52]	0.36
Monthly Income	1.02 ± 0.09	[0.85 1.22	0.80	1.18 ± 0.16	[0.91 1.53]	0.21
Health Status	1.86 ± 0.60	[1.01 3.41	0.05*	3.59 ± 1.56	[1.53 8.43]	0.003**
**Assessment of Satisfaction**						
**Factors**	**Odd Ratio**	**95% C.I**	**P**	**Odd Ratio**	**95% C.I**	**P**
Gender	1.30 ± 0.34	[0.76 2.20]	0.33	1.32 ± 0.48	[0.65 2.68]	0.44
Age group	1.09 ± 0.12	[0.89 1.35]	0.40	1.38 ± 0.25	[0.97 1.95]	0.07
Marital Status	1.03 ± 0.04	[084 1.12]	0.52	1.00 ± 0.06	[0.90 1.12]	0.98
Education	1.86 ± 0.63	[0.97 3.60]	0.06	0.92 ± 0.40	[0.39 2.17]	0.85
Monthly Income	1.03 ± 0.10	[0.86 1.25	0.69	1.00 ± 0.14	[0.75 1.32]	0.98
Health Status	1.83 ± 0.60	[0.97 3.47]	0.06	1.97 ± 0.87	[0.83 4.68]	0.12

*SE* Standard errors, *CI* Confidence interval, P-Value: *p* < 0.00***, *p* < 0.01**, *p* < 0.05*

### Qualitative data analysis findings

#### Changes principal members would like to make

In response to the open-ended question: “If you were a board member of your medical scheme, what three changes would you like to see?” The respondents’ answers can be summarized into three sub-themes, cost, benefits, and information.

### Cost of services

Regarding the cost of services, the main suggestion was that a GP should bill at a standard rate. Respondents also suggested that medical schemes should allocate more funds for medication, lower the annual contribution, and offer a rebate for consumers who have not used their medical aid for two years. Some respondents expressed the desire for a low membership contribution, a strategy to tackle waste and fraud in the medical scheme’s environment and more transparency with regards to DSPs.

#### Benefits

Regarding benefits, respondents expressed their desire to have more benefits without co-payments. Some suggested more PMB consultations, increase in GP and medical specialist consultations especially more dental and optometric benefits. A broader DSP network, outsourcing the right DSP, a full cover of prescribed drugs and an easy payment of claims were desired by some respondents. Some respondents expressed a need for benefit options suitable for pensioners.

#### Information

Respondents suggested more transparency when the schemes provided information to their members. The respondents suggested that communication in terms of benefits should be consistent with what the schemes offered, and that no relevant information should be hidden. Some participants expressed a desire that schemes provide concise information package, use of videos explaining certain aspects of the schemes, define a clear dispute resolution mechanism and offer better communication between schemes and their members.

## Discussion

The Medical Schemes Act, No. 131 of 1998 provides legal protection for medical schemes members [7, 11]. This study provides evidence on perceived knowledge of open scheme members and their satisfaction with their medical schemes.

The overall results showed that approximately 71% of participants felt that they had good knowledge of their medical scheme, compared to 54% who scored above 61% for satisfaction. This finding is consistent with the Health Market Inquiry (HMI) report which showed a strong correlation between good perceived knowledge and good satisfaction [6]. Seventy-five percent of the participants also understood the benefits and cost involved before they joined their scheme. The top four chronic diseases in participants were asthma, cardiac failure, hypertension, and thyroid conditions, consistent with the Council for Medical Schemes (CMS) data and with other studies conducted in South Africa on the burden of non-communicable disease [7, 13, 14]. The increased incidence of non-communicable diseases in South Africa is a public health concern and has consequences for medical scheme membership costs.

Thirty-eight percent of the respondents earned a monthly income of R30 000 (2 160 US$) or above. This raises the issue of affordability of medical schemes in South Africa which has a GDP/ capita of 6 281 USD and a high GINI coefficient. Kaplan highlighted the same concern in his findings [15]. McLeod and Ramjee pointed out that affordability constituted a major barrier to medical aid access in South Africa and was the greatest obstacle to the growth of the medical schemes industry [3]. Furthermore, the cost of entry-level medical schemes options remains mostly unaffordable [3]. Very few members (14%) were on a high-level option as members selected their option not based on their income or health needs, but on paying less, except when faced with a chronic disease.

The Healthcare consumer survey conducted in South Africa in 2016 found that 92% of participants who used brokers felt that they received satisfactory information [10]. This finding is higher than ours where only 62% of respondents perceived their brokers to be knowledgeable about medical schemes. Additionally, our study found that there was no relationship between good perceived knowledge and joining a medical scheme through brokers.

The finding of an association between perceived knowledge and income is consistent with Adewole’s study in Nigeria [15]. This may imply that members with better income are more able to access various sources of information that could improve their knowledge. Inconsistent with Adewole’s study [15], we found that the level of perceived knowledge and satisfaction was not associated with gender or suffering from a chronic disease. This dissimilarity may be due to a different study population and study setting. Adewole study’s population comprised mostly of farmers, artisans, and traders in a rural area, whereas South African open scheme members live mostly in urban areas. Ackah and Owusu in Ghana showed that older individuals were more knowledgeable in health insurance [12].

The PMBs are critical aspects of medical schemes regulation in South Africa, introduced after exclusions and high costs resulting from cream skimming and risk rating by the industry. Though almost half of the respondents indicated that they had made out of pocket payments for services, they were still satisfied with their medical schemes. Our study is limited, as it does not determine the amount of money spent out of pocket. We also did not know if the members were reimbursed or not or if the OOPs were the result of the use of non-Designated Service Provider GP or drugs not covered under the PMBs. The question of OOPs and satisfaction regarding PMBs needs further exploration. According to Mohammed, Sambo, and Dong, Odeyemi and Nixon, out of pocket payments (OOPs) had a negative impact on consumers as it delayed health access and promoted the use of alternative treatment [9, 16]. They suggested that OOPs were related to patient dissatisfaction [9, 16].

Some respondents expressed the desire to tackle waste and fraud in the medical scheme’s environment and the need for more transparency with regards to the DSP contract. The prevalence of health care fraud in South Africa is estimated at 5% to 15% of the total health care expenditure [17]. In South Africa, fraud waste and abuse adds approximately R22 billion (US$1,584 million) to the annual cost of private health care [18] in addition to other cost drivers [19].

The respondents also indicated their wish to have access to a comprehensive benefits package at an affordable rate without co-payment. These desires are echoed in the new Medical Schemes Amendment Bill, which suggested a comprehensive benefits package fully covered by the schemes and the abolition of the PMBs [20].

The Medical Schemes Act promotes DSP arrangements between medical schemes and healthcare providers to ensure proper service delivery of the PMBs [4, 7, 11]. To reduce the cost of health care services, many medical schemes have contracted with DSPs. Although anecdotal evidence shows that consumers do not like DSPs, this study showed that 81% of participants were satisfied with the quality of care received from the DSPs. However, the proportion of participants who had a good perceived knowledge of DSPs was only 55%. Eighty-one percent of participants did not have access to DSPs hospitals where they lived, while 9% were satisfied with access to a designated GP. This could be because of the location of the hospitals and GP or the cap on the number of consultations allowed. However, other unknown reasons may explain this limited access. In 2010, another healthcare survey found that 60% of medical schemes members had a negative attitude towards DSPs. In contrast to ours, this study did not evaluate the quality of service received from the DSPs, but rather, participants’ freedom of choice as they wanted to choose their own doctors as they found DSPs to be inconvenient [21].

Regarding complaints against medical schemes, the results showed that there was a statistically significant relationship between perceived knowledge and complaints. This emphasises the responsibility of the schemes to ensure that principal members have the necessary knowledge to get the most value out of their benefit option. Few participants made appeals against their medical schemes and the CMS. This is consistent with the study of Rodwin that showed that most schemes members chose not to appeal, even when they had a reasonable cause [22]. Respondents were quite satisfied when they appealed to their schemes, but dissatisfied with CMS outcome.

### Study limitation

The main limitation of this study was that to maintain confidentiality, the research team was not in direct contact with the interviewees and relied on the schemes to distribute the letters that linked to the URL of the questionnaire. Therefore, the researchers could not contact individuals who did not respond and had to rely on general reminders to the entire sample.

This study also used a cross-sectional survey. Our analysis could therefore not determine any underlying issues or infer a causal relationship. Recall bias could also be an issue for respondents who had a poor experience with their service providers or those who had not used their medical scheme recently.

## Conclusion

Medical schemes in South Africa provide financial risk protection for more than 8,8 million beneficiaries. This study showed that open medical scheme members felt that they had good knowledge of their medical schemes. Participants were however not satisfied with accessing DSPs, scheme contributions and the PMBs. The results therefore highlight the need for further development of the PMBs. The current PMBs review should offer a comprehensive benefits basket without co-payment for medical schemes members, as recommended by the Medical Schemes Act Amendment Bill [20]. These findings may be used as a patient’s satisfaction baseline under the NHI. It may also be useful to the medical schemes by pointing out the need to improve accessibility to hospitals, GP coverage, an extension of DSPs networks and the PMBs package [23, 24].

## Supplementary Information

**Additional file 1.** Survey of members’ perceived knowledge and satisfaction with medical schemes.

## Data Availability

The datasets used and/or analysed during the current study are available from the corresponding author on reasonable request.

## Abbreviations

PMBs: Prescribed minimum benefits
DSPs: Designated Service Providers
OOP: Out-Of-Pocket payments
KAP: Knowledge, Attitudes and Perceptions
