# Supplementary material for: Perceived knowledge of scheme members and their satisfaction with their medical schemes: a cross-sectional study in South Africa
Source: BMC Public Health. 2022 Sep 8;22:1700. doi: 10.1186/s12889-022-14106-8 (PMC9454120; doi:10.1186/s12889-022-14106-8)
Supplement: Supplementary file 1 — Additional file 1. Survey of members’ perceived knowledge and satisfaction with medical schemes. [file 12889_2022_14106_MOESM1_ESM.docx]

# SURVEY OF MEMBERS’ PERCEIVED KNOWLEDGE AND SATISFACTION WITH MEDICAL SCHEMES

Please tick on your answer using the space provided

**I. Medical scheme membership**

1. How many years have you been a member of any medical scheme? ________

2. Have you ever changed your medical scheme?

1 Yes

2 No

3. If yes, how many years have you been a member of your current medical scheme? _____

4. Medical schemes have different options which have different names. Based on the monthly premium for your scheme, which option are you currently on?

1 Low level option.

2 Medium level option

3 High level option

| 5. How would you rate your understanding of the benefits and costs involved before you joined your current medical scheme?  1 Not understood at all  2 Not understood  3 Not sure  4 Understood  5 Very well understood |
| --- |

6. Who advised you about the benefits and cost of the different medical schemes? (Please tick all appropriate options)

1 Employer

2 Broker

3 Medical Scheme’s Advisor

4 My own research

5 Other reasons (Please specify) ____________________

7. Which factors had influenced your choice of the benefits option? (Please tick all appropriate options)

1 Knowledge about the benefits package of the option

2 Cost

3 Other (Please specify) ____________________

8. Considering the benefits against the costs, what were the main reasons for you choosing the option you are on?

Please explain ______________________________________________________

9. Please rate the relevance of the information provided to you before joining your current medical scheme

1 Not at all relevant

2 Not so relevant

3 In the middle

4 Relevant

5 Very relevant

**II. General experience of the medical scheme**

10. It is easy to interact with my medical scheme

1 Strongly disagree

2 Disagree

3 Neither agree nor disagree

4 Agree

5 Strongly agree

11. Satisfaction score of the service provided by my medical scheme

1 Very dissatisfied

2 Dissatisfied

3 Neither satisfied nor dissatisfied

4 Satisfied

5 Very satisfied

**III. Brokers**

12. I joined a medical scheme through a broker

1 Yes

2 No

13. If yes, how satisfied are you with your broker

1 Very dissatisfied

2 Dissatisfied

3 Neither satisfied nor dissatisfied

4 Satisfied

5 Very satisfied

14. If yes to question 12. My broker is very knowledgeable about the medical schemes.

1 Strongly disagree

2 Disagree

3 Neither agree nor disagree

4 Agree

5 Strongly agree

**IV. Benefit Options**

15. Have you ever changed your medical scheme option?

Yes

No

16. If yes, why did you change your medical scheme option?

1 The premiums were too expensive

2 Broker advised me to change

3 Other (Please specify)----------------------------------------------------------------------------------------------------------------------------------------------------------------------------------------------

**V. Prescribed minimum benefits (PMBs)**

17. Have you heard about the Prescribed minimum benefits (PMBs)?

1 Yes

2 No

18.Your knowledge of the Prescribed Minimum Benefits

1No knowledge at all

2 Poor knowledge

3 Moderate knowledge

4 Good knowledge

5 Excellent knowledge

19. Satisfaction score of the package of the current Prescribed Minimum Benefits

0 Not applicable to me

1 Very dissatisfied

2 Dissatisfied

3 Neither satisfied nor dissatisfied

4 Satisfied

5 Very satisfied

20. Drug prescription coverage is adequate under my current PMBs plan.

0 Not applicable to me

1 Strongly disagree

2 Disagree

3 Neither agree nor disagree

4 Agree

5 Strongly agree

21. Consultations with General Practitioners (GP) coverage are adequate under my current medical scheme.

1 Strongly disagree

2 Disagree

3 Neither agree nor disagree

4 Agree

5 Strongly agree

22. Consultations with specialists are adequately covered under my current medical scheme.

1 Strongly disagree

2 Disagree

3 Neither agree nor disagree

4 Agree

5 Strongly agree

23. The financial contributions I make to my medical cost through (premiums, deductibles, co-insurance, etc.) are affordable.

1 Strongly disagree

2 Disagree

3 Neither Agree nor disagree

4 Agree

5 Strongly agree

24. Under the current PMBs, I have been requested to pay extra money for my treatment or drug.

1 Yes

2 No

**VI. Designated service providers (Doctors, hospitals contracted by the medical scheme to cover the full medical costs or to lower co-payments)**

25. I know who the designated service providers for my medical scheme are.

 Strongly disagree

 Disagree

 Neither agree nor disagree

 Agree

 Strongly agree

26. Please, score the quality of the service that you receive from a designated service provider.

 Never used one

 Poor quality

 Fair quality

 Good quality

 Very good quality

 Excellent quality

27. Belonging to my medical scheme enables me to access a doctor where I live.1 Strongly disagree

2 Disagree

3 Neither agree nor disagree

4 Agree

5 Strongly agree

28. Belonging to my medical scheme enables me to access a hospital where I live.

 Strongly disagree

 Disagree

 Neither agree nor disagree

 Agree

 Strongly agree

29. Belonging to my medical scheme enables me to access a doctor when needed.

 Strongly agree

 Agree

 Neither agree nor disagree

 Disagree

 Strongly disagree

30. Belonging to my medical scheme enables me to access a hospital when needed.

 Strongly agree

 Agree

 Neither agree nor disagree

 Disagree

 Strongly disagree

31. I am able to get an appointment with a specialist when needed.

 Strongly agree

 Agree

 Neither agree nor disagree

 Disagree

 Strongly disagree

**VII. Complaints and appeals**

32. Have you ever lodged a complaint with your scheme?

1 Yes

2 No

33. If yes, how satisfied were you with the outcome?

1 Very dissatisfied

2 Dissatisfied

3 Neither satisfied nor dissatisfied

4 Satisfied

5 Very satisfied

34. Have you lodged a complaint with the Council for Medical Scheme (CMS) before?

1 Yes

2 No

35. If yes, how satisfied were you with the outcome?

1 Very dissatisfied

2 Dissatisfied

3 Neither satisfied nor dissatisfied

4 Satisfied

5 Very satisfied

36. How likely are you to recommend your medical scheme to others?

1 Extremely unlikely

2 Unlikely

3 Neutral

4 Likely

5 Extremely likely

37. If you were a Board member of a medical scheme, what are the 3 changes you would like to see? (Please, separate your response by a comma or ideally put 1, 2, 3 and spaces on your response).

**VIII. Please tell us a little bit about yourself (Socio-demographic information)**

38. Gender

1 Male

2 Female

39. Age (Years): ____________________

40. Marital status

1 Married

2 Divorced

3 Widow

4 Single

4 Others (Please specify)

41. Level of education

1 No formal schooling

2 Primary school (Grade 1 to Grade 7)

3 Secondary School (Grade 8 to Grade 12)

4 Tertiary

| 42. What is your monthly income level? |  |
| --- | --- |

1 Less than R5000

2 R5000 and less than R10 000

3 R10 000 and less than R15 000

4 R15 000 and less than R25 000

5 R25 000 and less than R30 000

6 R30 000 and more

43. Score of your overall health status

1 Poor health

2 Somewhat healthy

3 Moderately healthy

4 Healthy

5 Excellent health

44. Do you suffer from any chronic disease?

 Yes

 No

45. If yes, please specify (Tick all applicable)

1 Diabetes

2 Hypertension (High blood pressure)

3 Asthma

3 Cardiac Failure

4 Rheumatoid arthritis

5 Other (Specify)……………….

**We thank you for your participation**
